# miR-1260b inhibits periodontal bone loss by targeting ATF6β mediated regulation of ER stress

**DOI:** 10.3389/fcell.2022.1061216

**Published:** 2022-11-30

**Authors:** Chikako Hayashi, Takao Fukuda, Kentaro Kawakami, Masaaki Toyoda, Yuki Nakao, Yukari Watanabe, Takanori Shinjo, Tomomi Sano, Misaki Iwashita, Karen Yotsumoto, Miyu Shida, Takaharu Taketomi, Terukazu Sanui, Takeshi Uchiumi, Takashi Kanematsu, Fusanori Nishimura

**Affiliations:** ^1^ Department of Periodontology, Division of Oral Rehabilitation, Faculty of Dental Science, Kyushu University, Fukuoka, Japan; ^2^ Department of Cell Biology, Aging Science, and Pharmacology, Division of Oral Biological Sciences, Faculty of Dental Science, Kyushu University, Fukuoka, Japan; ^3^ Dental and Oral Medical Center, Kurume University School of Medicine, Fukuoka, Japan; ^4^ Department of Clinical Chemistry and Laboratory Medicine, Graduate School of Medical Sciences, Kyushu University, Fukuoka, Japan

**Keywords:** miR-1260b, periodontitis, ER stress, GMSCs, osteoclast, ATF6β

## Abstract

The expression profiles of exosomal microRNAs (miRNAs) are regulated by the microenvironment, and appropriate priming with mesenchymal stem cells (MSCs) is one of the strategies to enhance the paracrine potency of MSCs. Our previous work demonstrated that exosomes from tumor necrosis factor (TNF)-α-primed human gingiva-derived MSCs (GMSCs) could be a therapeutic tool against periodontitis, and that TNFα-inducible exosomal miR-1260b is essential for the inhibition of alveolar bone loss. However, the precise molecular mechanism underlying miR-1260b-mediated inhibition of osteoclastogenesis is not yet fully understood. Here, we found that the activating transcription factor (*ATF*)-*6β*, a novel miR-1260b-targeting gene, is critical for the regulation of osteoclastogenesis under endoplasmic reticulum (ER) stress. An experimental periodontal mouse model demonstrated that induction of ER stress was accompanied by enhanced ATF6β expression, and local administration of miR-1260b and ATF6β siRNA using polyethylenimine nanoparticles (PEI-NPs) significantly suppressed the periodontal bone resorption. In periodontal ligament (PDL) cells, the ER stress inducer, tunicamycin, enhanced the expression of the receptor activator of NF-κB ligand (RANKL), while miR-1260b-mediated downregulation of ATF6β caused RANKL inhibition. Furthermore, the secretome from miR-1260b/ATF6β-axis-activated PDL cells inhibited osteoclastogenesis in human CD14^+^ peripheral blood-derived monocytes. These results indicate that the miR-1260b/ATF6β axis mediates the regulation of ER stress, which may be used as a novel therapeutic strategy to treat periodontal disease.

## Introduction

Dental tissue-derived mesenchymal stem cells (MSCs) have gained attention because of the advantages of easy acquisition *via* routine dental procedures and superior outcomes in both regenerative and immunoregulatory therapeutics (Orsini et al., 2018) (Li et al., 2021). Recent studies have reported the therapeutic potential of the MSCs-derived secretome, which includes trophic factors such as cytokines, growth factors, and extracellular vesicles (EVs) (El Moshy et al., 2020) (Pethő et al., 2018). EVs act as mediators of intercellular communication, and are broadly classified into microvesicles (100–1,000 nm), exosomes (40–100 nm), and apoptotic bodies (1–5 μm) (Raposo and Stoorvogel, 2013). Exosomes possess the ability to transfer genetic information through RNAs and proteins, thereby inducing phenotypic changes in recipient cells (Quesenberry et al., 2015). Specifically, exosomal microRNA (miRNA)-mediated modification of cellular phenotypes plays a central role in the therapeutic effects of MSC-derived exosomes (Valadi et al., 2007). Interestingly, growing evidence has suggested that appropriate preconditioning of MSCs with disease-related stimuli can optimize the contents of exosomes to efficiently support the repair of specific diseases (Katsuda and Ochiya, 2015).

Periodontitis is the most common osteolytic inflammatory disease caused by specific periodontal disease-causing bacteria (Hajishengallis, 2015). Its pathology is characterized by inflammation of the periodontium and subsequent destruction of the tooth-supporting alveolar bone, which is a major cause of tooth loss in adults (Kajiya and Kurihara, 2021). Recently, studies have focused on the influence of endoplasmic reticulum (ER) stress on periodontitis (Jiang et al., 2022). ER is the principal organelle that plays an essential role in the folding and structural maturation of proteins. ER stress is characterized by the activation of the unfolded protein response (UPR) under conditions, such as oxidative stress, inflammation, and aging (Hetz et al., 2020). UPR is mediated by three major ER transmembrane proteins: double-stranded RNA-dependent protein kinase-like ER kinase, inositol-requiring transmembrane kinase and endonuclease 1, and activating transcription factor 6 (ATF6). UPR protein upregulation activates transcription factors, including nuclear factor-κB and activator protein-1, leading to the expression of pro-inflammatory cytokines (Garg et al., 2012). Furthermore, ER stress causes alveolar bone loss following oral infection with *Porphyromonas gingivalis* in mice (Yamada et al., 2015). These findings strongly indicate that activated ER stress exacerbates the destruction of periodontal tissue. However, a therapeutic strategy for periodontal disease that targets the MSC-derived exosomal miRNA-mediated regulation of ER stress has not yet been reported.

Among dental tissue-derived MSCs, human gingiva-derived MSC (GMSCs) have a distinct neural crest origin and exhibit some advantages over other MSCs (Zhang et al., 2017). Human GMSCs exhibit prominent immunomodulatory and proliferative capacities with stable functional characteristics at high passage numbers (Kim et al., 2021). Importantly, both mouse and human GMSCs secrete large amounts of exosomes than other somatic MSCs (Kou et al., 2018). Based on a strategy to improve the anti-inflammatory properties of MSC-derived exosomes by priming MSCs with cytokines (Park et al., 2018), we recently demonstrated that the tumor necrosis factor (TNF)-primed human GMSC-derived exosomes significantly enhanced the therapeutic effects in a mouse periodontal model (Nakao et al., 2021). Although we further found that the TNF-inducible exosomal miR-1260b is a critical regulator of periodontal bone loss, the detailed mechanism has not yet been fully elucidated.

In this study, we first screened novel miR-1260b-targeting genes *via* database analysis and found that they could be associated with ER stress by targeting ATF6β. There are two closely related forms of ATF6, ATF6α (670 aa) and ATF6β (703 aa), which have structural similarities (Thuerauf et al., 2007). While most studies have focused on ATF6α and its role in modulating ER stress, the role of ATF6β is poorly understood. Therefore, we investigated the therapeutic effects of miR-1260b on osteoclastogenesis in periodontal disease by targeting ATF-mediated ER stress.

## Materials and methods

### Bioinformatic analysis

The target genes of human miR-1260b were predicted using the database TargetScan (total context + + score <−0.7) (https://www.targetscan.org/vert_80/) and mirDIP (Integrated Score > 0.6 and score class was set to “very high” (top 1%)) (https://ophid.utoronto.ca/mirDIP/).

### Mice

C57BL/6NCrSlc mice (female, 8-week-old) were purchased from Japan SLC (Hamamatsu, Japan) and used under an institutionally approved animal research protocol (protocol #A21-131-2; Kyushu University).

### Histology, immunohistochemistry and immunofluorescence staining

Maxillae were removed and fixed in 4% paraformaldehyde for 24 h. Thereafter, samples were demineralized using ethylenediaminetetraacetic acid solution (Osteosoft; Merck, Darmstadt, Germany) for 72 h and embedded in paraffin. Standard hematoxylin and eosin staining and dual-color immunofluorescence analysis using specific primary antibodies for mouse anti-ATF6β antibody (1:100, 15794-1-AP; Proteintech) were performed, as previously described (Nakao et al., 2021). Images were captured using a confocal laser-scanning microscope (Carl Zeiss LSM 700; Oberkochen, Germany) and ZEN 2012 software.

### Preparation of miR-1260b or ATF6β siRNA-polyethylenimine nanoparticles and fluorescent labelling of miRNA

Linear PEI-NPs (in vivo-jetPEI) were purchased from Polyplus-Transfection SA (Illkirch-Graffenstaden, France). miRCURY LNA microRNA mimic for hsa-miR-1260b or control miRNA (negative control: cel-miR-39-3p; Qiagen, Hilden, Germany) was dissolved in 5% glucose solution at a concentration of 50 μM. Stealth™ RNAi duplexes against mouse ATF6β, a mixture of three different siRNAs (MSS236243, MSS236245 and MSS273801) or Stealth™ RNAi negative control duplex (Invitrogen Life Technologies, Carlsbad, CA, United States) were dissolved in 5% glucose solution at a concentration of 20 μM. Solutions containing miRNA or siRNA and PEI-NPs were mixed and incubated for 15 min at room temperature. A total of 10 μl of miR-PEI-NPs (miR: 125 pmol) or siRNA-PEI-NPs (siRNA: 50 pmol) was injected. The distribution of miRNA in mice was tracked using Cy-3 labeling miRNA. miR-1260b was labeled with the Label IT siRNA Tracker Cy3 Kit without any transfection reagent (Mirus Bio LLC., WI, United States).

### Ligature-induced periodontal model in mice

Experimental periodontitis was induced as previously described (Nakao et al., 2021). Mice were randomly divided into the following three groups: 1) placebo (*in vivo* jetPEI only), 2) miR-control, and 2) miR-1260b mimic. To induce periodontal bone loss in mice, a 5-0 silk ligature (Akiyama Medical MFC Co., Tokyo, Japan) was tied around the right maxillary second molar. After ligation, the placebo or miR-PEI-NPs were injected into the palatal gingiva of the maxillary second molar using a 33-gauge needle Hamilton syringe (Hamilton Company. NV, United States).

### Micro-computed tomography scanning

The samples were scanned using a Micro-computed tomography (micro-CT) imaging system (ScanXmate; Comscan, Kanagawa, Japan). The measurement conditions for micro-CT included tube voltage (kV), electrical current (A), image voxel size, and slice thickness. After scanning, three-dimensional (3D) images were reconstructed using TRI/3D-BON software (Ratoc System Engineering, Tokyo, Japan). To evaluate alveolar bone resorption, bone resorption volumes were superimposed on the non-ligated and ligated sides. The differences between the non-ligated and ligated sides were measured by following the selection of a 3D region of interest as described below. Crown buccal-palatal widths were defined as the buccal-palatal landmark. M1’s palatal root apex and M2 were assigned as the mesial-distal landmarks. The cement–enamel junction and M1 and M2 palatal root apexes were defined as tooth axial landmarks (Supplementary Figure S1A). Moreover, (non-ligated–ligated) bone volume/non-ligated bone volume (%) was taken as the degree of bone resorption. The alveolar bone loss of each group was also defined as the sum of distances from the eight sites as indicated in Supplementary Figure S1B.

### Cytokines and reagents

Recombinant human macrophage colony-stimulating factor (M-CSF), receptor activator of NF-κB ligand (RANKL) and mouse RANKL were purchased from BioLegend (San Diego, CA, United States). Tunicamycin was obtained from Cayman Chemical Co. (Ann Arbor, MI, United States).

### Cell culture

Human CD14^+^ peripheral blood-derived monocytes (PBMCs) were purchased from Lonza (Basel, Switzerland). The cells were cultured in the Roswell Park Memorial Institute 1,640 medium (Nacalai Tesque, Kyoto, Japan) supplemented with 10% heat-inactivated fetal bovine serum, 2 mM glutamine (Nacalai Tesque), 1% sodium pyruvate (Nacalai Tesque), 1% non-essential amino acids (Nacalai Tesque), and 25 ng/ml M-CSF. Human periodontal ligament (PDL) cells (Lonza) were grown in complete fibroblast medium (FM) using the FibroLife S2 Comp Kit (Kurabo Industries Ltd., Osaka, Japan), and cells at the second to third passages were used for subsequent experiments. The mouse osteoclast precursor clone RAW-D cells, which were derived from RAW264 macrophage-like cell line were kindly gifted by Prof. Toshio Kukita and Prof. Takayoshi Yamaza (Kyushu University, Japan) and cultured as previously described (Watanabe et al., 2004) (Kukita et al., 2004). RAW-D cells were stimulated with 50 ng/ml RANKL to differentiate into osteoclasts.

### Quantitative RT-PCR analysis

Quantitative RT-PCR (qRT-PCR) was performed as previously described (Watanabe et al., 2022). Total RNA was isolated from the cells using ISOGENⅡ (Nippon Gene, Tokyo, Japan), and first-strand cDNA was synthesized using PrimeScript RT Master Mix (Takara Bio, Otsu, Japan). qRT-PCR was performed using the Luna Universal qPCR Master Mix (NEW ENGLAND BioLabs Inc.) on a StepOnePlus Real-Time System (Applied Biosystems, Carlsbad, CA, United States) under the following conditions: 95°C for 1 min, 40 cycles of 95°C for 15 s, and 60°C for 30 s. Primer sequences used in this study are listed in Supplementary Table S1 **.**

### Western blot analysis

Western blotting was performed as previously described (Watanabe et al., 2022). Cells were washed with phosphate-buffered saline and lysed in Passive Lysis 5× buffer (Promega, Madison, WI, United States). Nuclear proteins were extracted using a LysoPure Nuclear and Cytoplasmic Extractor Kit (FUJIFILM, Tokyo, Japan). Protein samples were separated on polyacrylamide gels and transferred to polyvinylidene difluoride membranes. The membranes were incubated with the appropriate primary antibodies, anti-ATF6β (1:1000; 15794-1-AP, Proteintech, Rosemont, IL, United States), anti-RANKL (1:1000; 12A668, Novus Biologicals, United States), anti-CHOP (1:1000, L63F7, Cell Signaling Technology), anti-Grp78 (1:1000, C50B12, Cell Signaling Technology), anti-cleaved caspase-3 (1:1000, 5A1E, Cell Signaling Technology), anti-JNK (1:1000, 56G8, Cell Signaling Technology), anti-Phospho-JNK (Thr183/Tyr185) (1:1000, 81E11, Cell Signaling Technology), anti-Lamin B1 (1:1000, 1298-1-AP, Proteintech) and anti-β-actin (1:1000; 13E5, Cell Signaling Technology) antibodies, and secondary antibodies, anti-rabbit IgG (1:2000; Cell Signaling Technology) and anti-mouse IgG (1:2000; Cell Signaling Technology). Blotted membranes were visualized using a densitometry technique on Amersham ImageQuant 800 (Cytiva, Tokyo, Japan) and quantified using Multi Gauge 3.1 software (FUJIFILM, Tokyo, Japan).

### Cell transfection *in vitro*

Transfection of miRNA mimics and siRNAs for PDL cells was performed using the Lipofectamine RNAiMAX Transfection Reagent (Thermo Fisher Scientific. MA, United States) for 24 h according to our reverse transfection protocol (Fukuda et al., 2013). For knockdown analysis, Stealth™ RNAi duplexes against human ATF6β, a mixture of three different siRNAs (HSS102271, HSS102272 and HSS102273) and mouse ATF6β, a mixture of three different siRNAs (MSS236243, MSS236245 and MSS273801) were obtained from the Invitrogen Corporation (Invitrogen Life Technologies). Stealth™ RNAi negative control duplex (Medium GC Duplex, Invitrogen Life Technologies) was used as a control. For miRNA mimic transfection, PDL cells were transfected with 20 pmol miRCURY LNA microRNA mimic for hsa-miR-1260b (Qiagen) or control miRNA (negative control: cel-miR-39-3p; Qiagen). RAW-D cells were transfected with mouse ATF6β siRNAs or control siRNA using Hiperfect Transfection Reagent (Qiagen), according to the manufacturer’s protocol.

### Dual-luciferase reporter assay

The fragment of ATF6β 3′-UTR (WT) and its corresponding mutated sequence (MUT) designed by converting the miR-1260b binding sequence 5′-GGUGGGA to 5′-ACTCAA, were synthesized and cloned into the pmirGLO dual-luciferase miRNA Target Expression Vector (Promega, Madison, WI, United States), termed ATF6β-3′-UTR-WT (5′-AAA​CTA​GCG​GCC​GCT​AGA​GGG​TGG​T) and ATF6β-3′-UTR-MUT (5′-AAA​CTA​GCG​GCC​GCT​AGT​AAC​TCA​T), respectively. Reporter plasmids containing the WT or MUT ATF6β 3′-UTR (0.5 μg) and miR-1250b mimic or control miRNA (20 pmol) were co-transfected into PDL cells using Lipofectamine 3000 (Thermo Fisher Scientific. MA, United States). After 24 h of incubation, cells were lysed. Firefly and Renilla luciferase activities were detected using a dual-luciferase assay system (Promega, WI, United States).

### WST-8 viability assay

PDL cells (5 × 10^3^ cells) in 100 µl of FM were seeded into a 96-well culture plate in triplicate with or without 0.5 μg/ml of tunicamycin. The number of seeded cells was limited to ensure that cell growth would continue until the end of incubation period, without reaching cell confluence. Next, 10 µl of WST-8 solution (Cell Count Reagent SF™: Nakarai Tesuque, Kyoto, Japan) were added to each well (including the control wells), at 3, 6, 12, 24 and 48 h. Cells were incubated for an additional 1 h at 37°C, then absorbance at 450 nm was measured, with a reference reading at 650 nm.

### Tartrate-resistant acid phosphatase staining and osteoclast quantification

CD14^+^ PBMCs (5 × 10^4^) were placed in a 96-well plate. The cells were incubated with RANKL (30 ng/ml) and M-CSF (25 ng/ml) for 2 weeks with or without tunicamycin treatment for the first 3 days. The medium was replaced every 3 days. Cultured cells were fixed with 10% formalin for 5 min and then with ethanol–acetone (50:50 vol/vol) for 1 min at room temperature. Tartrate-resistant acid phosphatase (TRAP) staining was performed using a TRAP/ALP Stain Kit (FUJIFILM Wako). Photographs were taken using BZ8000 (Keyence Co., Osaka, Japan). The number of TRAP-positive cells was counted for each number of nuclei (7 or 10 ≤ nuclei) (Piper et al., 1992). Areas of each TRAP-positive cell were measured using the ImageJ software (version 1.43; National Institute of Health, Bethesda, MD, United States).

### Pit resorption assay

CD14^+^ PBMCs were cultured at a density of 1 × 10^5^ cells/well in a bone resorption assay plate 48 (PG Research, Tokyo, Japan) coated with calcium phosphate (CaP-coated). The cells were incubated as described above. After 14 days, the CaP-coated plate was treated with 5% sodium hypochlorite (Sigma-Aldrich, St Louis, Missouri, United States) for 5 min, according to the manufacturer’s instructions. The resorption pit areas were analyzed using the ImageJ software.

### Statistical analysis

Data are presented as the mean ± standard deviation. Data analyses were performed using GraphPad Prism 9 (GraphPad Software Inc., La Jolla, CA, United States). One- or two-way analysis of variance followed by correction for multiple comparisons with Tukey’s post hoc test were used to compare three or more groups to analyze the statistical significance. Other statistical comparisons of data from the two groups were performed using the Student’s *t*-test. Statistical significance was set at *p* < 0.05.

## Results

### Identification of ATF6β as a novel miR-1260b target gene and increased ATF6 expression in a mouse periodontal model

**FIGURE 1:**
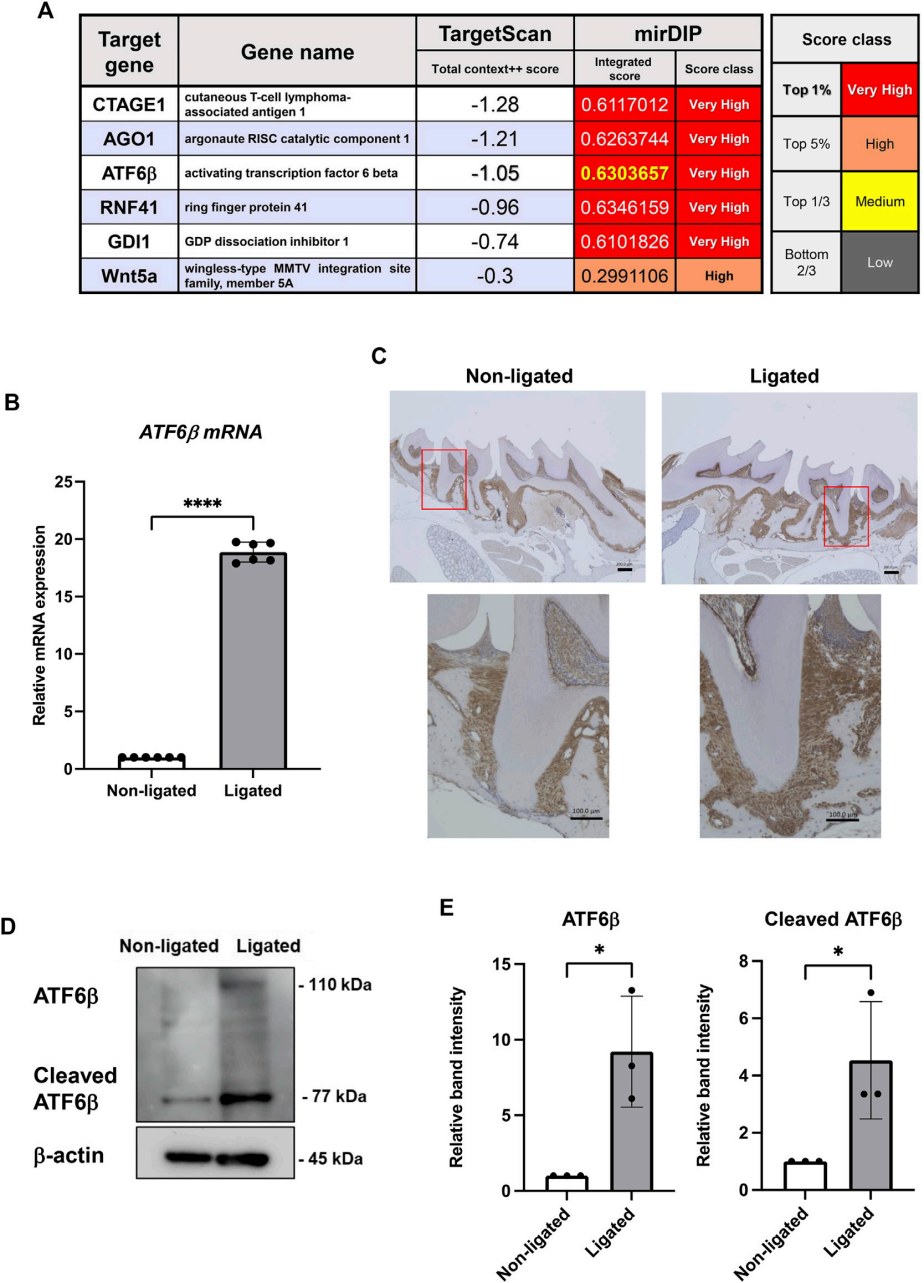
Expression levels of the activating transcription factor (*ATF*)-*6β*, a novel microRNA (miR)-1260b-targeting gene, are upregulated in the mice periodontal model. **(A)** TargetScan and miRDIP databases were used to identify novel top target genes of miR-1260b. **(B–E)** Enhanced expression of ATF6β in ligature-induced mice periodontal model. A 5-0 silk ligature was tied around the maxillary second molar in C57BL/6 mice for 7 days. **(B)** Relative mRNA expression of ATF6β in mice gingival tissue (*n* = 6). **(C)** Immunohistochemical staining of ATF6β in mice periodontal tissue. **(D)** Western blotting analysis revealed the expression levels of full-length and cleaved ATF6β in mice gingiva, and the relative expression was measured (*n* = 3). β-actin was used as the control. **(D)**. *p < 0.05, ****p < 0.0001. Error bars represent the mean ± standard deviation (SD). The significance of differences between groups was determined using one-way Tukey’s test.

To explore the most critical gene for biological regulation by miR-1260b in periodontal tissue, miRDIP (https://ophid.utoronto.ca/mirDIP/) and TargetScan (https://www.targetscan.org/vert_80/) databases were analyzed. First, we selected the top five overlapping TargetScan (total context + + score <−0.7) and mirDIP (Integrated Score >0.6) genes (Figure 1A). Considering that *ATF6β* is an ER stress response-associated gene and emerging evidence has indicated the involvement of ER stress in periodontal disease, we focused on ATF6β in this study. In a mouse periodontitis model, the expression of ATF6β mRNA was upregulated in ligated-gingival tissue (Figure 1B). Immunohistochemical staining revealed that ATF6β was mainly expressed in both the PDL and subgingival connective tissue, and the accumulated expression of ATF6β was observed along the alveolar bone resorption area (Figure 1C). Enhanced protein expression of ATF6β in the inflammatory periodontium was confirmed by western blotting analysis (Figures 1D,E).

### Local injection of miR-1260b suppresses periodontal bone loss in mice

**FIGURE 2:**
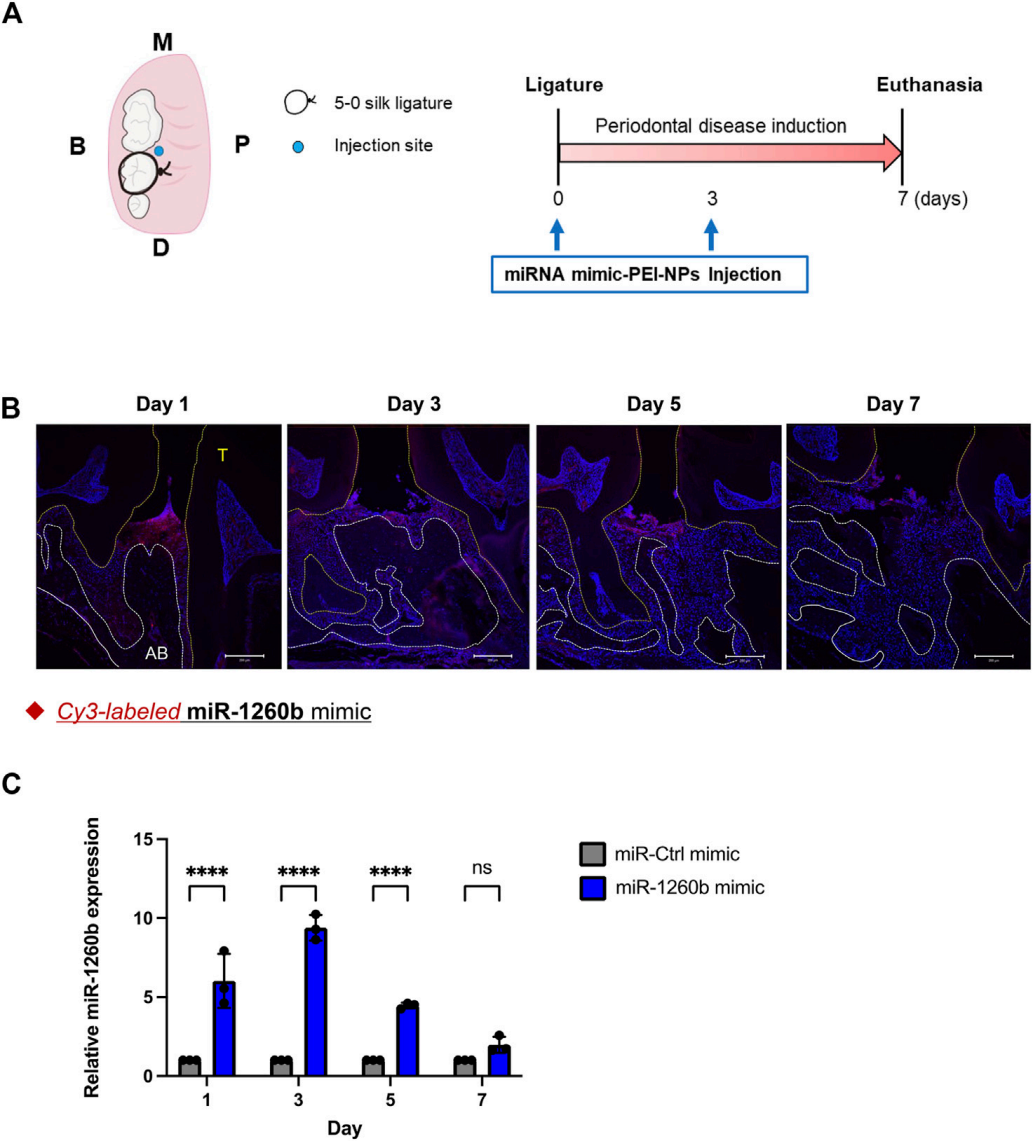
Distribution of locally injected miR-1260b mimic-polyethylenimine nanoparticles (PEI-NPs) in the mice periodontal model. **(A)** Schematic illustration of the ligature-induced periodontitis model and local administration of miRNA-PEI-NPs. A 5-0 silk ligature was tied around the maxillary second molar in C57BL/6 mice on day 0. On day 0 and 3, a total of 10 μl of miR-PEI-NPs (miR: 125 pmol) was injected as illustrated. **(B)** Detection of injected miRNA in mice periodontal tissue. miRNAs were Cy-3 labeled (Red) before administration. Nuclei were stained with 4, 6-diamino-2-phenylindole (DAPI; blue). AB: alveolar bone, T, teeth. Scale bar = 200 μm. **(C)** Time course expression of miR-1260b in mice gingiva (*n* = 3). ns, not significant, ****p < 0.0001. Error bars represent the mean ± SD. The significance of differences between groups was determined using two-way analysis of variance (ANOVA), followed by correction for multiple comparisons with Tukey’s post hoc test.

**FIGURE 3:**
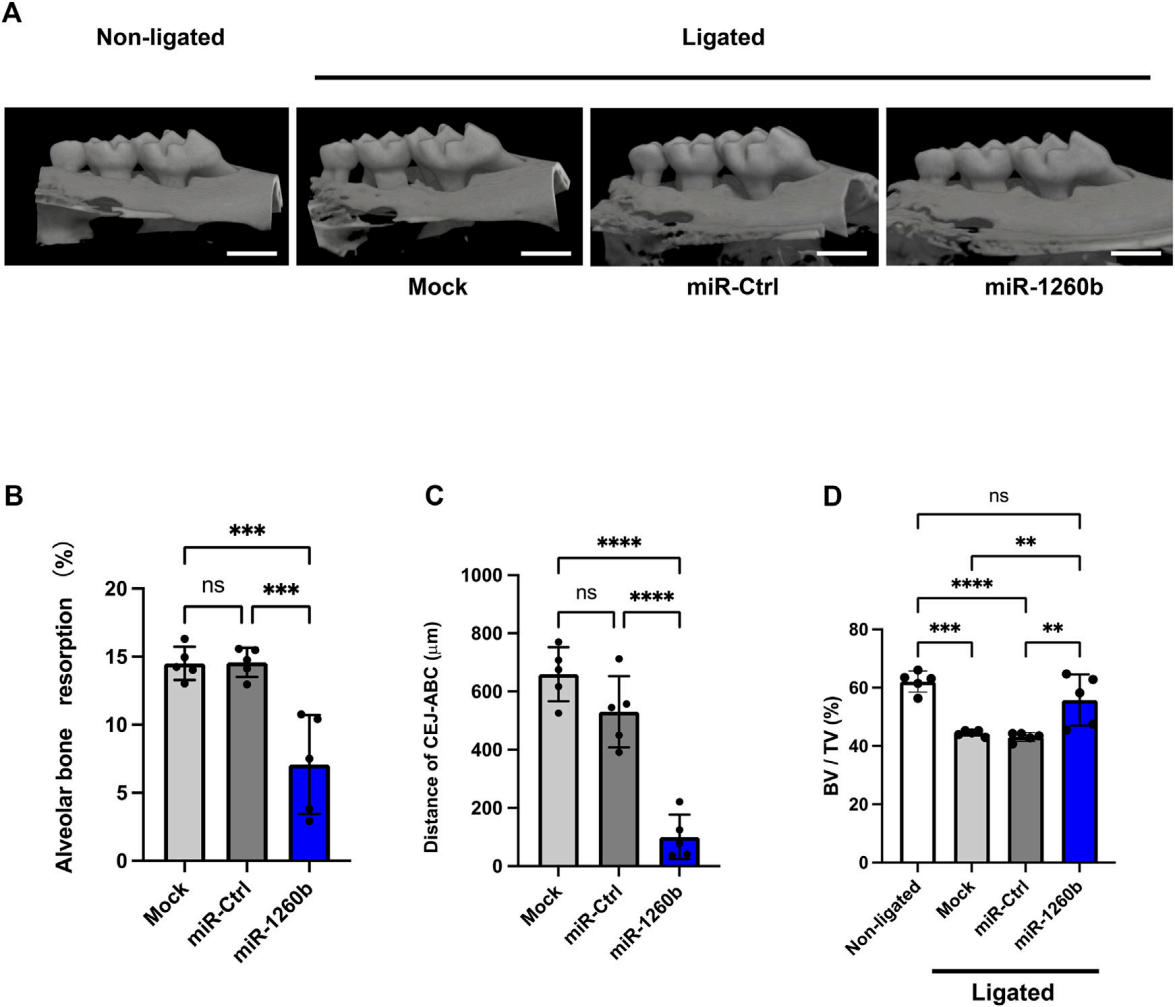
Local injection of miR-1260b inhibits alveolar bone loss in the mice periodontal model. **(A)** Three-dimensional micro-computed tomography (micro-CT) images of the maxillae in each treatment group on day 7 after ligature placement. Scale bar = 1,000 μm. **(B–D)** Periodontal bone resorption analysis was performed using a split-mouth experimental design: one side of the maxilla was ligated and locally injected with miR-mimic-PEI-NPs, whereas the other side without ligation served as its own control. **(B,C)** Relative alveolar bone resorption volume **(B)** and the distance from the cementoenamel junction (CEJ) to the pinnacle of the alveolar bone (AB) **(C)** were calculated by (non-ligated–ligated) groups. **(D)** Bone volume/tissue volume (BV/TV). Error bars represent the mean ± SD, *n* = 6. ns: not significant, **p < 0.01, ***p < 0.001, ****p < 0.0001. The significance of differences between groups was determined using one-way ANOVA, followed by correction for multiple comparisons with Tukey’s post hoc test.

**FIGURE 4:**
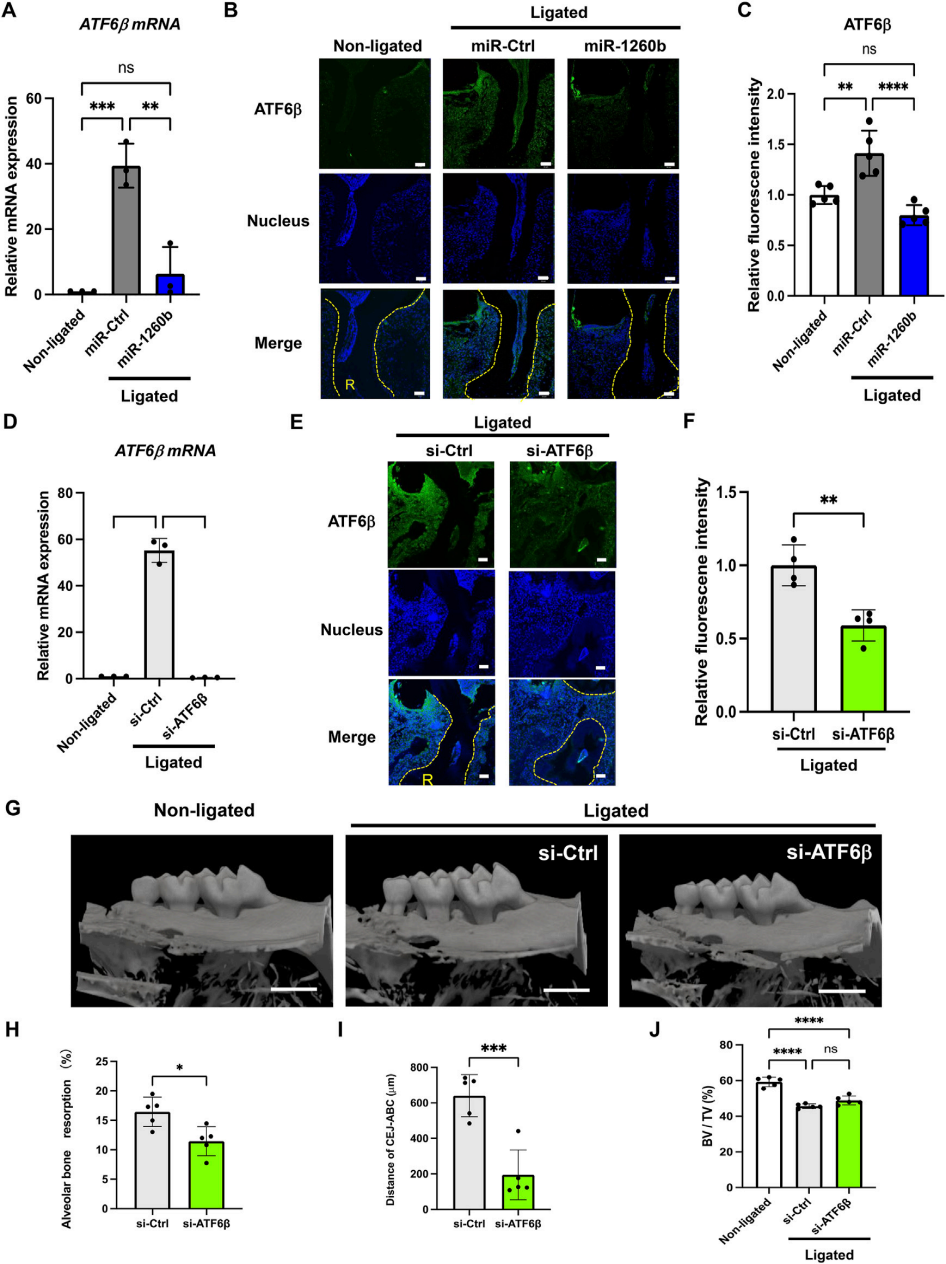
miR-1260b-mediated down-regulation of ATF6β is essential for the inhibition of periodontal bone loss. Effects of miRNA injection on the expression of ATF6β in the mice periodontal model on day 1 **(A)** and day 7 **(B,C)** after ligation. **(A)** ATF6 β mRNA expression in mouse gingival tissue (*n* = 3). **(B)** Immunofluorescence staining of ATF6β (green) in the mouse periodontal tissue. Nuclei were stained with DAPI (blue). R, root. Scale bar = 50 μm. **(C)** Intensity of ATF6β fluorescence in each sample was measured in each group (*n* = 5). **(D–F)** Validation of ATF6β knockdown in mice periodontal model on day 1 **(D)** and day 7 **(E,F)** after ligation. **(D)** ATF6 β mRNA expression in mouse gingival tissue (*n* = 3). **(E)** Immunofluorescence staining of ATF6β (green) in the mouse periodontal tissue. Nuclei were stained with DAPI (blue). R, root. Scale bar = 50 μm. **(F)** Intensity of ATF6β fluorescence in each sample was measured in each group (*n* = 4). **(G)** Three-dimensional micro-computed tomography (micro-CT) images of the maxillae in each treatment group on day 7 after ligature placement. Scale bar = 1,000 μm. **(H–J)** Periodontal bone resorption analysis was performed using a split-mouth experimental design: one side of the maxilla was ligated and locally injected with siRNA-PEI-NPs, whereas the other side without ligation served as its own control. **(H,I)** Relative alveolar bone resorption volume and **(H)** the distance from the cementoenamel junction (CEJ) to the pinnacle of the alveolar bone (AB) **(I)** were calculated by (non-ligated–ligated) groups. **(J)** Bone volume/tissue volume (BV/TV). Error bars represent the mean ± SD, *n* = 6. ns, not significant, **p < 0.01, ***p < 0.001, ****p < 0.0001. The significance of differences between groups was determined using one-way ANOVA, followed by correction for multiple comparisons with Tukey’s post hoc test.

To investigate the inhibitory and therapeutic potential of miR-1260b against ligature-induced periodontal bone loss *in vivo*, a miR-1260b mimic was injected into the interdental region of second molar (M2) using PEI-NPs (Figure 2A). Relative alveolar bone resorption was determined using a split-mouth experimental design, in which one side of the maxilla (upper jaw) was ligated and locally injected with miR mimic-PEI NPs, whereas the other side was not ligated. Thus, each animal served as a control. Fluorescent imaging revealed that Cy3-labeled miR-1260b mimic-PEI-NPs were retained in the interdental gingiva for 7 days (Figure 2B). qRT-PCR further confirmed the local administration-induced upregulation of miR-1260b expression in the gingival tissue (Figure 2C). Compared with the non-ligated site, severe alveolar bone loss was observed around the ligated M2 in both the mock- and miR-Ctrl-injected groups. In contrast, local injection of the miR-1260b mimic significantly reduced the bone resorption on day 7 after ligation (Figure 3). To gain further insight into miR-1260b-mediated inhibition of ATF6β, the expression level of ATF6β in periodontal tissue was monitored. qRT-PCR analysis revealed that treatment with miR-1260b significantly inhibited the ligation-induced expression of ATF6β mRNA (Figure 4A). Consistently, immunofluorescence staining also demonstrated the downregulated expression of ATF6β by the miR-1260b mimic on day 7 after ligation (Figures 4B,C). To validate that miR-1260b-mediated downregulation of ATF6β is responsible for the inhibition of periodontal bone loss, ATF 6β siRNA-PEI NPs was injected in mice periodontal model. qRT-PCR and immunofluorescence staining confirmed the knockdown of ATF6β expression in mice periodontal tissue (Figures 4D–F). Micro-CT analysis demonstrated that ATF 6β siRNA-PEI NPs significantly reduced alveolar bone resorption than Ctrl-siRNA-injected groups (Figures 4G–I). On the other hand, there was no difference between Ctrl and ATF6β siRNA injected groups for BV/TV (Figure 4J), indicating that the inhibitory effect for bone loss by miR-1260b is stronger than ATF6β siRNA. Collectively, these results suggest that the miR-1260b-mediated downregulation of ATF6β is responsible for the inhibition of ligation-induced alveolar bone loss.

### miR-1260b attenuates the nuclear expression of ATF6β to suppress receptor activator of NF-κB ligand expression in periodontal ligament cells

**FIGURE 5:**
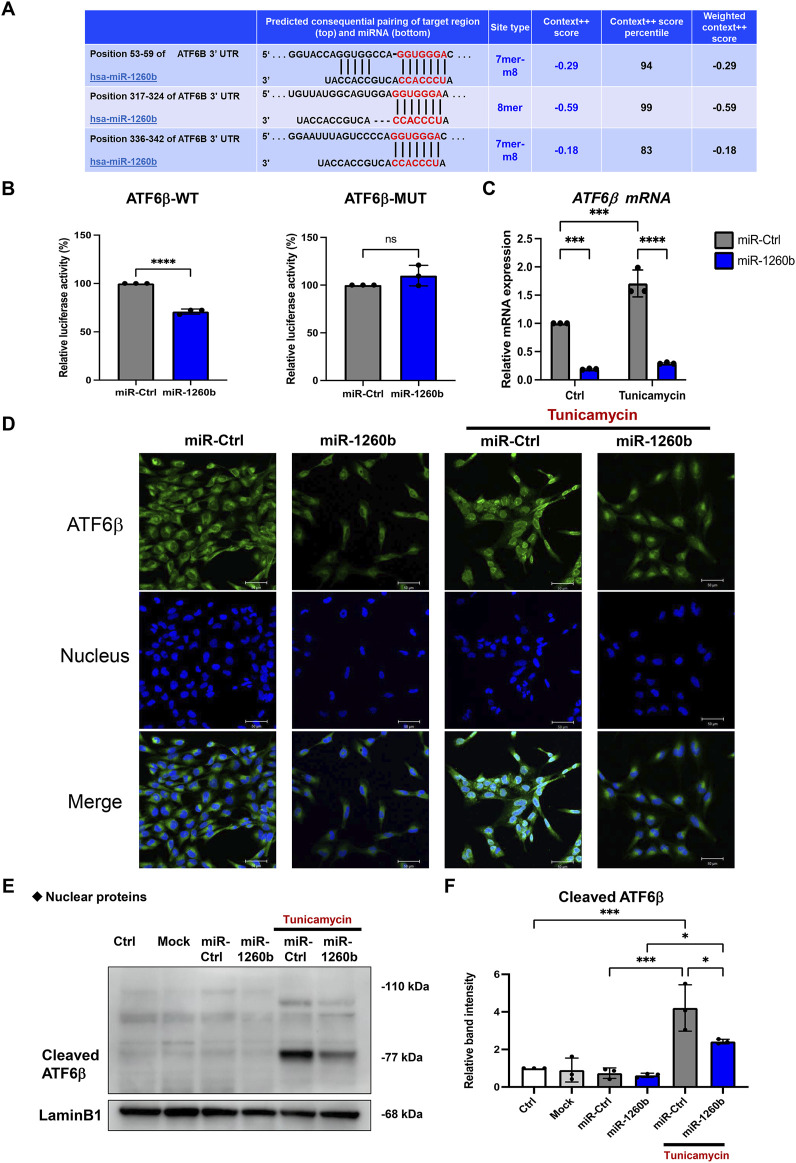
miR-1260b attenuates the endoplasmic reticulum (ER) stress-induced nuclear expression of cleaved ATF6β in periodontal ligament (PDL) cells. **(A)** Binding sites (in red letters) between ATF6β 3′-untranslated region (UTR) and miR-1260b predicted on the TargetScan database. **(B)** Luciferase reporter vectors containing the wild type miR-1260b binding sites of ATF6β (ATF6β-WT) or mutated ATF6β sequence (ATF6β-Mut) were co-transfected with control miRNA (miR-Ctrl, 20 nM) or miR-1260b mimic (20 nM) into human primary PDL cells. After 24 h, firefly luciferase activity in each sample was measured and normalized to the control (Renilla) luciferase activity. **(C–E)** Effect of miR-1260b on the expression of ATF6β under ER stress. PDL cells were transfected with miR-Ctrl or miR-1260b for 24 h. After changing the medium, cells were stimulated with 0.5 μg/ml of tunicamycin for 24 h. **(C)** Expression levels of ATF6β mRNA were measured *via* quantitative reverse transcription-polymerase chain reaction (qRT-PCR). **(D)** Representative confocal images of the cellular localization of ATF6β expression (green). Nuclei were stained with DAPI (Blue). Scale bar = 50 μm. **(E,F)** Nuclear fraction proteins were isolated to analyze the expression of nuclear translocated cleaved-ATF6β and the relative expression was measured (*n* = 3). Lamin B1 was used as the control for nuclear protein. ns, not significant, *p < 0.05, ***p < 0.001, ****p < 0.0001. The significance of differences between groups was determined using two-way ANOVA, followed by correction for multiple comparisons with Tukey’s post hoc test.

**FIGURE 6:**
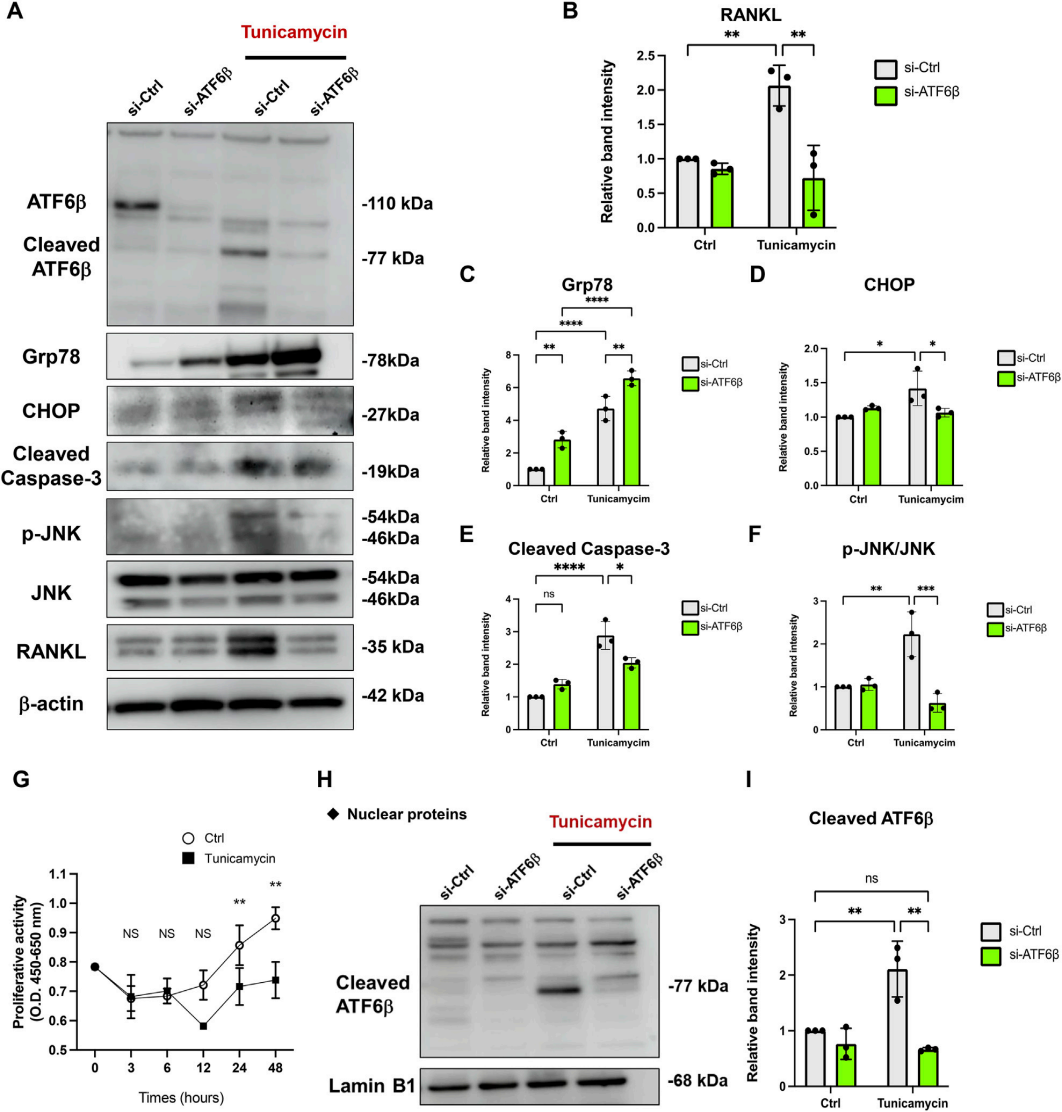
Knockdown of ATF6β suppresses the endoplasmic reticulum (ER) stress-induced RANKL expression in periodontal ligament (PDL) cells. Effect of ATF6β-siRNA on the expression of RANKL under ER stress. PDL cells were transfected with si-Ctrl or si-ATF6β for 24 h. After changing the medium, cells were stimulated with 0.5 μg/ml of tunicamycin for 24 h **(A–F)** Western blotting analysis revealed the expression levels of full-length/cleaved ATF6β, Grp78, CHOP, Cleaved Caspase-3, phospho-JNK (p-JNK), JNK and RANKL in PDL cells and the relative expression was measured (*n* = 3). β-actin was used as the control. **(G)** The effect of 0.5 μg/ml of tunicamycin on proliferation of PDL cells was measured using a WST-8 assay. Proliferation was measured at the indicated time points (shown in the X-axis) and expressed as (absorbance at 450 nm)—(absorbance at 655 nm) (*n* = 3). **(H,I)** Nuclear fraction proteins were isolated to analyze the expression of nuclear translocated cleaved-ATF6β and the relative expression was measured (*n* = 3). Lamin B1 was used as the control for nuclear protein. ns, not significant, **p < 0.01. The significance of differences between groups was determined using one-way ANOVA, followed by correction for multiple comparisons with Tukey’s post hoc test.

Next, we explored the molecular mechanism of miR-1260b-mediated ATF6β suppression in the inhibition of bone resorption. Putative binding sites for miR-1260b within the 3′-UTR sequence of ATF6β were identified using TargetScan (Figure 5A). As the PDL–alveolar bone interface plays a critical role in periodontal bone homeostasis (Kanzaki et al., 2001), we performed a dual-luciferase reporter assay to confirm the physical binding of miR-1260b to ATF6β mRNA in primary PDL cells. Inserts harboring the predicted ATF6β WT (ATF6β-WT) and MUT (ATF6β -Mut) sequences were cloned into a luciferase reporter vector. Co-transfection of ATF6β-WT with the miR-1260b mimic in PDL cells showed a significant decrease in the relative luciferase activity compared to that in the negative control miRNA mimic (NC-miR mimic) group. This was not observed in the experiments using ATF6β-Mut (Figure 5B). This confirmed the preferential binding of miR-1260b to the 3′-UTR of ATF6β. Next, we assessed the miR-1260b-mediated downregulation of ATF6β expression under ER stress. Treatment of PDL cells with the ER stressor, tunicamycin, significantly increased the expression of ATF6β mRNA (Figure 5C), whereas the induction of ATF6β was diminished *via* transfection with the miR-1260b mimic. ATF6β is proteolytically cleaved during the ER stress response to release cleaved ATF6β (Thuerauf et al., 2007). As a transcription factor, the cleaved active form of ATF6β is translocated to the nucleus. Immunofluorescence staining captured by confocal microscopy demonstrated that tunicamycin-induced potent nuclear accumulation of ATF6β was diminished by miR-1260b (Figure 5D). Western blotting further confirmed that tunicamycin-mediated ER stress increased the expression of cleaved ATF6β in the nuclear fraction of PDL cells, which was significantly inhibited by miR-1260b (Figures 5E,F). We subsequently examined whether ER stress-induced cleavage of ATF6β was involved in RANKL expression. ATF6β knockdown successfully inhibited the nuclear expression of cleaved ATF6β (Figures 6A,B), thereby abrogating tunicamycin-induced RANKL expression in PDL cells (Figures 6H,I). Tumnicamycin not only enhanced the expression of other ER stress-associated marker proteins for Grp78 (Figure 6C) and CHOP (Figure 6D), but also apoptosis marker for cleaved caspase-3 (Figure 6E). Knockdown of ATF6β down-regulated the expression of CHOP and cleaved caspase-3, while it up-regulated the expression of Grp78. Furthermore, tunicamyci-mediated phosphorylation of JNK was attenuated by ATF6β knockdown (Figure 6F). The treatment of PDL cells with 0.5 mg/ml of tunicamycin inhibited cell growth (Figure 6F). These results indicate that the ER stress-induced RANKL expression is regulated by ATF6β, and miR-1260b targets ATF6β to mediate RANKL inhibition in PDL cells.

### Secretome from the miR-1260b/ATF6β-axis-activated periodontal ligament cells suppresses osteoclastogenesis

**FIGURE 7:**
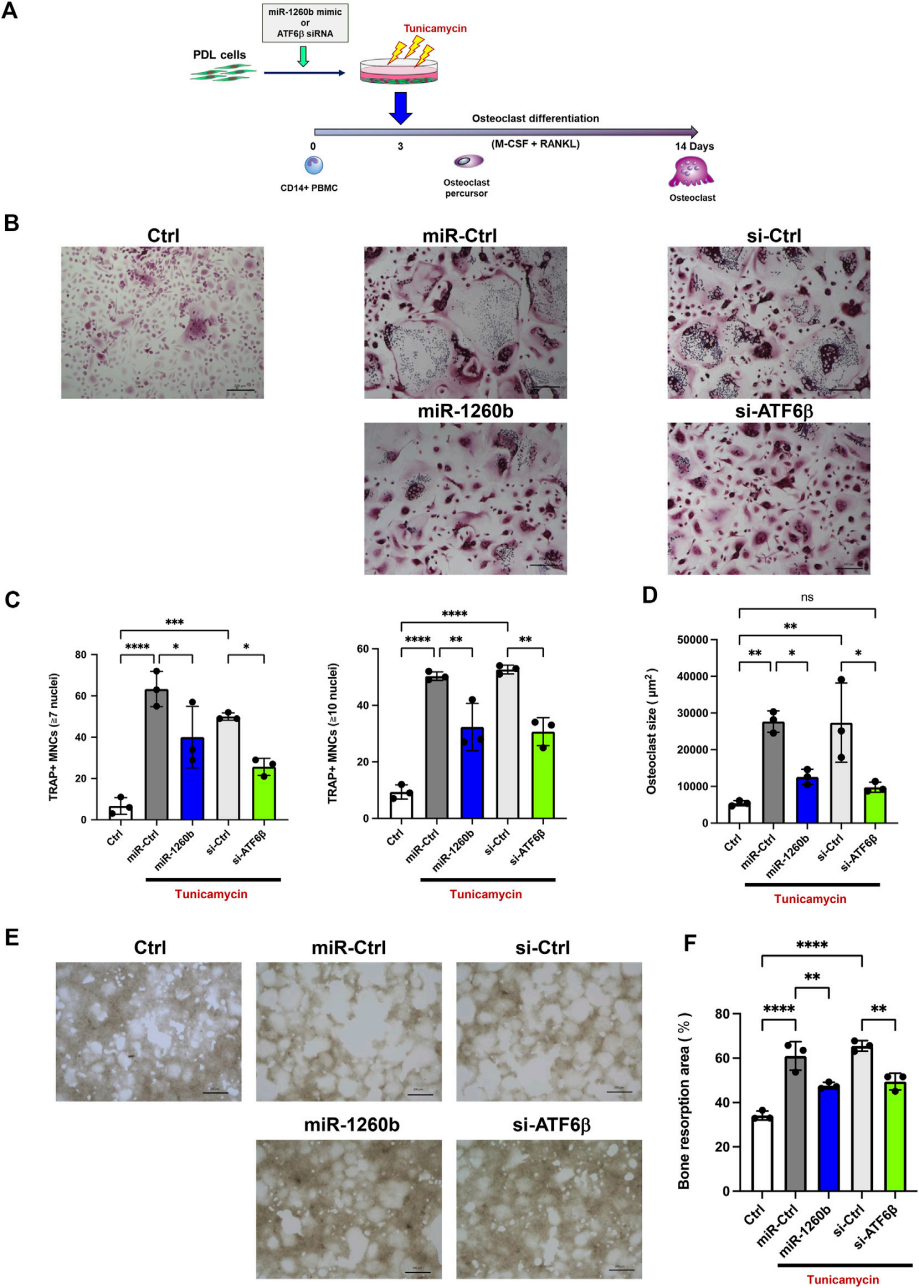
Cell culture supernatant from miR-1260b mimic/ATF6β small interfering RNA (siRNA)-transfected PDL cells inhibits osteoclastogenesis in CD14^+^ peripheral blood-derived monocytes (PBMCs). **(A)** Scheme for the validation of the effect of cell culture supernatant from PDL cells on osteoclast differentiation. PDL cells were transfected with either miRNA mimic (miR-Ctrl or miR-1260b) or siRNA (si-Ctrl or si-ATF6β) for 24 h. After changing the medium, cells were stimulated with tunicamycin (0.5 μg/ml) for 24 h. CD14^+^ PBMCs were stimulated with M-CSF for 3 days, and then incubated with the cell culture supernatant from PDL cells as described above for additional 3 days. PBMCs were further stimulated with the receptor activator of NF-κB ligand (RANKL)/macrophage colony-stimulating factor (M-CSF) for up to 14 days **(B)** Representative images of tartrate-resistant acid phosphatase (TRAP) staining. Scale bar = 200 μm. **(C,D)** Osteoclast differentiation was evaluated by the number of TRAP-positive multinucleated cells (MNCs) (7 or 10 ≤ nuclei) **(C)** and the size of TRAP-positive MNCs (3 ≤ nuclei) **(D)**. *n* = 3. **(E)** Representative images of the resorption pit on the calcium phosphate (CaP)-coated plate. Scale bar = 200 μm. **(F)** Total resorbed area in each culture was measured. *n* = 3. *p < 0.05, **p < 0.01, ****p < 0.0001. The significance of differences between groups was determined using one-way ANOVA, followed by correction for multiple comparisons with Tukey’s post hoc test.

We further evaluated the effects of RANKL regulation on osteoclastogenesis in PDL cells. TRAP staining revealed that incubation of human CD14^+^ monocytes with cell culture supernatant from tunicamycin-stimulated PDL cells enhanced the number and size of TRAP^+^ multinucleated mature osteoclasts. In contrast, incubation with miR-1260b mimic and ATF6β siRNA-transfected cell culture supernatant significantly inhibited osteoclast differentiation (Figures 7A–D). Bone resorption assays were performed to assess the activity of differentiated osteoclasts. Consistent with the results of TARP staining, osteoclasts differentiated from the cell culture supernatants of ER stress-induced PDL cells exhibited high bone resorption activity, which was diminished by incubation with miR-1260b mimic and ATF6β siRNA transfectants (Figures 7E,F).

## Discussion

The present study provides new insights into the molecular mechanisms of miR-1260b-mediated suppression of periodontal bone loss *via* the regulation of ER stress. To our knowledge, most studies have focused the effect of miR-1260b on cell proliferation or migration (Xia et al., 2020) (Seong and Kang, 2020). While we identified miR-1260b as a novel TNF-inducible GMSC-derived exosomal miRNA (Nakao et al., 2021), miR-1260b was reported to be downregulated in gingival tissue from periodontitis (Stoecklin-Wasmer et al., 2012). Thus, we speculated that the local administration of miR-1260b could contribute to the treatment of periodontal disease. Although we previously indicated that exosomal miR-1260b could inhibit RANKL expression by targeting Wnt5a (Nakao et al., 2021), the direct effect of miR-1260b on osteoclastogenesis, both *in vitro* and *in vivo*, has not yet been validated. As ATF6β is the top-ranked target of miR-1260b in multiple miRNA target database analysis (Figure 1A) and ER stress is involved in osteoclastogenesis (Collison, 2018), miR-1260b-mediated regulation of ER stress may be a potential target for the inhibition of alveolar bone loss.

In the present study, we demonstrated that miR-1260b mediated the downregulation of ATF6β. We also revealed that suppression of the nuclear accumulation of cleaved-ATF6β was indispensable for the inhibition of RANKL in PDL cells. ATF6 protein is proteolytically cleaved in response to ER stress. The N-terminus of ATF6 has a transcriptional activation domain, whereas the C-terminus has basic leucine zipper (Leu-Zip) and ER transmembrane (ERTM) domains. Under ER stress, serine proteases in the Golgi apparatus cleave the ERTM domains of ATF6 to release activated cleaved ATF6. The cleaved form of the ATF6 protein indicates that the UPR is activated (Thuerauf et al., 2007). While the effect of activated ATF6α-mediated inflammation has been well established (Stengel et al., 2020), a recent study further demonstrated the activation of ATF6β under ER stress conditions (Hien and Back, 2021). Indeed, we also observed increased expression of both full-length and cleaved active forms of ATF6β in a mouse periodontal model in this study (Figure 1).

In a ligature-induced mouse periodontal model, we confirmed that the local administration of miR-1260b successfully inhibited the alveolar bone loss (Figure 3), which was accompanied by attenuated ATF6β expression (Figure 4). To deliver the miR-1260b mimic in mice, we used the in vivo-jetPEI reagent, which has been previously used for gene delivery in *in vivo* studies and clinical trials with high stability and without any toxicity (Nezami et al., 2014). In the current study, immunofluorescence imaging and qRT-PCR analysis revealed that the miR-1260b mimic using in vivo-jetPEI reagent was successfully delivered to and sustained the gingival tissue (Figure 2). Although the expression of exogenously added miR-1260b was reduced after 7 days (Figure 2), ATF6β expression continued to be downregulated (Figure 4). In contrast, the expression of ATF6β was significantly upregulated in ligated gingiva injected with the miR-Ctrl mimic. Furthermore, local injection of ATF6β siRNA significantly suppressed periodontal bone loss in mice. These results demonstrate that both induction of ATF6β by ligation and inhibition of ATF6β by miR-1260b were established in the ligature-induced mouse periodontal model, and the miR-1260b-mediated downregulation of ATF6β in periodontal tissue is responsible for the inhibition of alveolar bone loss. Although the aim of the current study was to validate the direct effect of miR-1260b, exosomes have a double-membrane structure that provides exosomal miRNAs with high stability and resistance to degradation. Therefore, miR-1260b encapsulated in exosomes may be more stable and should be validated in future studies. Importantly, Cy3-labeled miR-1260b was not localized around the alveolar bone, suggesting that inhibition of osteoclastogenesis is indirectly mediated by teeth surrounding the periodontal tissue.

We further investigated the precise molecular mechanism of miR-1260b-mediated RANKL inhibition during ER stress. Since PDL cells are involved in the regulation of osteoclastogenesis in alveolar bone through the release of RANKL (Kanzaki et al., 2001), we used primary PDL cells in an *in vitro* study. Luciferase assay revealed site-specific miR-1260b regulation of ATF6β, which was further validated for endogenous ATF6β expression (Figures 5A–C). The suppression ratio of endogenous ATF6β was more significant than that of the luciferase activity. This could be due to the difference in the number of predicted binding sites for miR-1260b; the ATF6β-WT plasmid contained one binding site, whereas the 3′UTR of endogenous ATF6β contained three binding sites. In PDL cells, the tunicamycin-mediated nuclear accumulation of cleaved ATF6β was abolished by miR-1260b (Figures 5E,F). This indicated an inhibitory effect of miR-1260b on ER stress. Although the association between ER stress and periodontal disease has been well accepted (Jiang et al., 2022), the direct effect of ER stress on osteoclastogenesis remains unclear. Yamada et al. (2015) reported that the ER stress inducer, tunicamycin, did not induce osteoclast differentiation without RANKL. Accordingly, we demonstrated that tunicamycin-induced RANKL expression was inhibited by ATF6β downregulation in PDL cells (Figure 6). To induce ER stress in PDL cells, we used 0.5 μg/ml of tunicamycin throughout the study. Tunicamycin is known as a cell cycle arrest molecule (Brewer et al., 1999) and the inhibition of cell proliferation in PDL cells were confirmed (Figure 6G). Interestingly, knockdown of ATF6β enhanced the expression of Grp78 (Figure 6C). Since Grp78 retains ATF6 in regulating ER homeostasis (Shen et al., 2002), it is possible that the expression of Grp78 was increased to compensate the decreased ATF6β. It is widely accepted that ATF6β regulates apoptosis (Nakanishi et al., 2005), and the down-regulation of cleaved caspase-3 by ATF6β knockdown under tunicamycin stimulation (Figure 6E) could also contribute to inhibition of apoptosis (Xiong et al., 2017). Furthermore, knockdown of ATF6β attenuated the tunicamycin-induced activation of JNK (Figure 6F). As the JNK signaling has reported to regulate RANKL expression (Luo et al., 2006) (Xiang et al., 2014), ATF6β-mediated inactivation of JNK could contribute to the inhibition of RANKL in PDL cells. On the other hand, we confirmed that knockdown of ATF6β had no effect on osteoclast differentiation markers expression (Supplementary Figure S2). Thus, we speculated that the miR-1260b/ATF6β axis mediated the regulation of RANKL in PDL cells and could influence the differentiation of osteoclasts. As revealed in our results, tunicamycin-treated cell culture supernatant from PDL cells increased mature osteoclast formation and activity of PBMCs, while those from miR-1260b-overexpressed or ATF6β-knockdown PDL cells significantly diminished these effects (Figure 7). Taken together, our data suggest that the interaction between PDL cells and osteoclasts is involved in the regulation of ER stress-induced periodontal bone resorption.

One limitation of this study is that the effect of miR-1260b on the secretome of PDL cells could not be evaluated. Since we are currently focusing on RANKL expression, proteomic analysis should be performed in future studies to understand the comprehensive expression profiles of other osteoclast-inducible cytokines, such as the monocyte chemotactic protein-1 (Kim et al., 2006) (Andrukhov et al., 2017). Another limitation is that the source of RANKL is not restricted to the PDL cells in periodontal tissues (Chen et al., 2014). However, the possible regulation of PDL cell-mediated osteoclastogenesis by miR-1260b contributes to our understanding of the molecular basis of ER stress in the pathogenesis of periodontitis.

**FIGURE 8:**
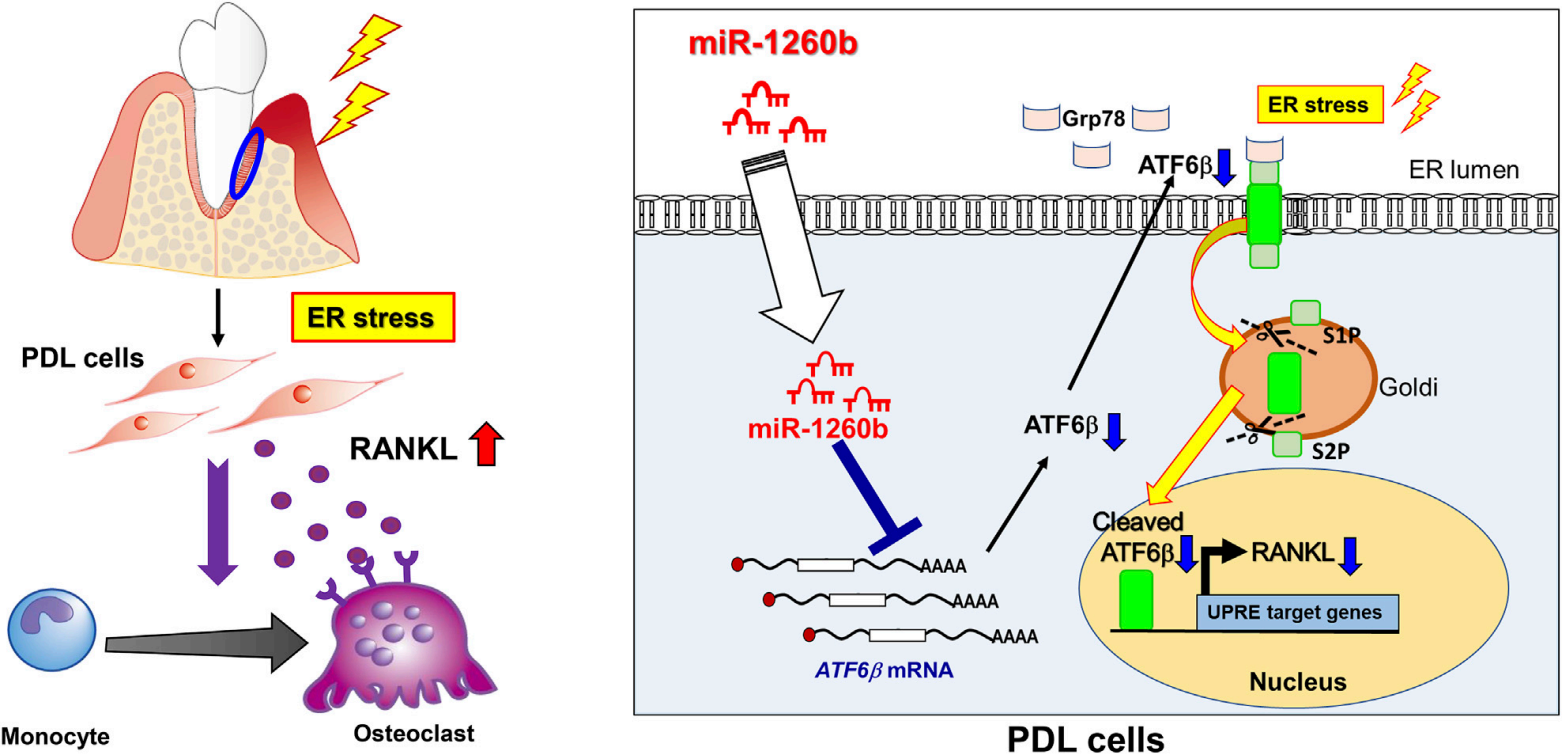
Therapeutic effects of miR-1260b on osteoclastogenesis in periodontal disease by targeting ATF6β-mediated ER stress. (left) Under pathological condition, ER stress is upregulated in periodontal tissue. PDL cells produce RANKL to stimulate osteoclast differentiation, which cause alveolar bone resorption. (right) In PDL cells, ATF6β binds to Grp78 and localize at ER lumen. During ER stress, ATF6β dissociate from Grp78 and cleaved by Site-1 protease (S1P) and Site-2 protease (S2P). The active from of cleaved-ATF6β translocate to nucleus and enhance RANKL expression in PDL cells. miR-1260b, a novel TNF-inducible GMSC-derived exosomal miRNA, targets ATF6β mRNA to downregulate RANKL expression, thereby inhibit osteoclastogenes in periodontal diseases.

In conclusion, our study demonstrated that TNF-inducible exosomal miR-1260b inhibits osteoclastogenesis *via* ATF6β-mediated regulation of ER stress. miR-1260b suppresses the nuclear expression of active ATF6β in PDL cells to downregulate RANKL expression, thereby contributing to the inhibition of periodontal bone loss. Our findings based on the molecular mechanisms are summarized in Figure 8. These findings are of considerable therapeutic significance to understand the cellular and molecular mechanisms of TNF-α-preconditioned GMSC-derived exosomes in periodontitis and other inflammatory disorders, eventually resulting in bone loss.

## Data Availability

The original contributions presented in the study are included in the article/Supplementary Material, further inquiries can be directed to the corresponding authors.

## References

[B1] Andrukhov O. Hong J. S. Andrukhova O. Blufstein A. Moritz A. Rausch-Fan X. (2017). Response of human periodontal ligament stem cells to IFN-γ and TLR-agonists. Sci. Rep. 7 (1), 12856. 10.1038/s41598-017-12480-7 28993635 PMC5634407

[B2] Brewer J. W. Hendershot L. M. Sherr C. J. Diehl J. A. (1999). Mammalian unfolded protein response inhibits cyclin D1 translation and cell-cycle progression. Proc. Natl. Acad. Sci. U. S. A. 96 (15), 8505–8510. 10.1073/pnas.96.15.8505 10411905 PMC17546

[B3] Chen B. Wu W. Sun W. Zhang Q. Yan F. Xiao Y. (2014). RANKL expression in periodontal disease: Where does RANKL come from? Biomed. Res. Int. 2014, 731039. 10.1155/2014/731039 24719884 PMC3955606

[B4] Collison J. (2018). Bone: ER stress causes osteoclastogenesis. Nat. Rev. Rheumatol. 14 (4), 184. 10.1038/nrrheum.2018.24 29467452

[B5] El Moshy S. Radwan I. A. Rady D. Abbass M. M. S. El-Rashidy A. A. Sadek K. M. (2020). Dental stem cell-derived secretome/conditioned medium: The future for regenerative therapeutic applications. Stem Cells Int. 2020, 7593402. 10.1155/2020/7593402 32089709 PMC7013327

[B6] Fukuda T. Sanui T. Toyoda K. Tanaka U. Taketomi T. Uchiumi T. (2013). Identification of novel amelogenin-binding proteins by proteomics analysis. PLoS One 8 (10), e78129. 10.1371/journal.pone.0078129 24167599 PMC3805512

[B7] Garg A. D. Kaczmarek A. Krysko O. Vandenabeele P. Krysko D. V. Agostinis P. (2012). ER stress-induced inflammation: Does it aid or impede disease progression? Trends Mol. Med. 18 (10), 589–598. 10.1016/j.molmed.2012.06.010 22883813

[B8] Hajishengallis G. (2015). Periodontitis: From microbial immune subversion to systemic inflammation. Nat. Rev. Immunol. 15 (1), 30–44. 10.1038/nri3785 25534621 PMC4276050

[B9] Hetz C. Zhang K. Kaufman R. J. (2020). Mechanisms, regulation and functions of the unfolded protein response. Nat. Rev. Mol. Cell Biol. 21 (8), 421–438. 10.1038/s41580-020-0250-z 32457508 PMC8867924

[B10] Hien L. T. Back S. H. (2021). Establishment of a reporter system for monitoring activation of the ER stress transducer ATF6β. Biochem. Biophys. Res. Commun. 558, 1–7. 10.1016/j.bbrc.2021.04.052 33894672

[B11] Jiang M. Li Z. Zhu G. (2022). The role of endoplasmic reticulum stress in the pathophysiology of periodontal disease. J. Periodontal Res. 57 (5), 915–932. 10.1111/jre.13031 35818935

[B12] Kajiya M. Kurihara H. (2021). Molecular mechanisms of periodontal disease. Int. J. Mol. Sci. 22 (2), 930. 10.3390/ijms22020930 33477754 PMC7832304

[B13] Kanzaki H. Chiba M. Shimizu Y. Mitani H. (2001). Dual regulation of osteoclast differentiation by periodontal ligament cells through RANKL stimulation and OPG inhibition. J. Dent. Res. 80 (3), 887–891. 10.1177/00220345010800030801 11379890

[B14] Katsuda T. Ochiya T. (2015). Molecular signatures of mesenchymal stem cell-derived extracellular vesicle-mediated tissue repair. Stem Cell Res. Ther. 6, 212. 10.1186/s13287-015-0214-y 26560482 PMC4642616

[B15] Kim D. Lee A. E. Xu Q. Zhang Q. Le A. D. (2021). Gingiva-derived mesenchymal stem cells: Potential application in tissue engineering and regenerative medicine - a comprehensive review. Front. Immunol. 12, 667221. 10.3389/fimmu.2021.667221 33936109 PMC8085523

[B16] Kim M. S. Day C. J. Selinger C. I. Magno C. L. Stephens S. R. Morrison N. A. (2006). MCP-1-induced human osteoclast-like cells are tartrate-resistant acid phosphatase, NFATc1, and calcitonin receptor-positive but require receptor activator of NFkappaB ligand for bone resorption. J. Biol. Chem. 281 (2), 1274–1285. 10.1074/jbc.M510156200 16280328

[B17] Kou X. Xu X. Chen C. Sanmillan M. L. Cai T. Zhou Y. (2018). The Fas/Fap-1/Cav-1 complex regulates IL-1RA secretion in mesenchymal stem cells to accelerate wound healing. Sci. Transl. Med. 10 (432), eaai8524. 10.1126/scitranslmed.aai8524 29540618 PMC6310133

[B18] Kukita T. Wada N. Kukita A. Kakimoto T. Sandra F. Toh K. (2004). RANKL-induced DC-STAMP is essential for osteoclastogenesis. J. Exp. Med. 200 (7), 941–946. 10.1084/jem.20040518 15452179 PMC2213286

[B19] Li B. Ouchi T. Cao Y. Zhao Z. Men Y. (2021). Dental-derived mesenchymal stem cells: State of the art. Front. Cell Dev. Biol. 9, 654559. 10.3389/fcell.2021.654559 34239870 PMC8258348

[B20] Luo X. H. Guo L. J. Xie H. Yuan L. Q. Wu X. P. Zhou H. D. (2006). Adiponectin stimulates RANKL and inhibits OPG expression in human osteoblasts through the MAPK signaling pathway. J. Bone Min. Res. 21 (10), 1648–1656. 10.1359/jbmr.060707 16995820

[B21] Nakanishi K. Sudo T. Morishima N. (2005). Endoplasmic reticulum stress signaling transmitted by ATF6 mediates apoptosis during muscle development. J. Cell Biol. 169 (4), 555–560. 10.1083/jcb.200412024 15897261 PMC2171703

[B22] Nakao Y. Fukuda T. Zhang Q. Sanui T. Shinjo T. Kou X. (2021). Exosomes from TNF-α-treated human gingiva-derived MSCs enhance M2 macrophage polarization and inhibit periodontal bone loss. Acta Biomater. 122, 306–324. 10.1016/j.actbio.2020.12.046 33359765 PMC7897289

[B23] Nezami B. G. Mwangi S. M. Lee J. E. Jeppsson S. Anitha M. Yarandi S. S. (2014). MicroRNA 375 mediates palmitate-induced enteric neuronal damage and high-fat diet-induced delayed intestinal transit in mice. Gastroenterology 146 (2), 473–483. 10.1053/j.gastro.2013.10.053 24507550 PMC3920196

[B24] Orsini G. Pagella P. Mitsiadis T. A. (2018). Modern trends in dental medicine: An update for internists. Am. J. Med. 131 (12), 1425–1430. 10.1016/j.amjmed.2018.05.042 29969611

[B25] Park W. S. Ahn S. Y. Sung S. I. Ahn J. Y. Chang Y. S. (2018). Strategies to enhance paracrine potency of transplanted mesenchymal stem cells in intractable neonatal disorders. Pediatr. Res. 83 (1-2), 214–222. 10.1038/pr.2017.249 28972960

[B26] Pethő A. Chen Y. George A. (2018). Exosomes in extracellular matrix bone Biology. Curr. Osteoporos. Rep. 16 (1), 58–64. 10.1007/s11914-018-0419-y 29372401 PMC5812795

[B27] Piper K. Boyde A. Jones S. J. (1992). The relationship between the number of nuclei of an osteoclast and its resorptive capability *in vitro* . Anat. Embryol. 186 (4), 291–299. 10.1007/bf00185977 1416078

[B28] Quesenberry P. J. Aliotta J. Deregibus M. C. Camussi G. (2015). Role of extracellular RNA-carrying vesicles in cell differentiation and reprogramming. Stem Cell Res. Ther. 6, 153. 10.1186/s13287-015-0150-x 26334526 PMC4558901

[B29] Raposo G. Stoorvogel W. (2013). Extracellular vesicles: Exosomes, microvesicles, and friends. J. Cell Biol. 200 (4), 373–383. 10.1083/jcb.201211138 23420871 PMC3575529

[B30] Seong M. Kang H. (2020). Hypoxia-induced miR-1260b regulates vascular smooth muscle cell proliferation by targeting GDF11. BMB Rep. 53 (4), 206–211. 10.5483/BMBRep.2020.53.4.136 31818357 PMC7196185

[B31] Shen J. Chen X. Hendershot L. Prywes R. (2002). ER stress regulation of ATF6 localization by dissociation of BiP/GRP78 binding and unmasking of Golgi localization signals. Dev. Cell 3 (1), 99–111. 10.1016/s1534-5807(02)00203-4 12110171

[B32] Stengel S. T. Fazio A. Lipinski S. Jahn M. T. Aden K. Ito G. (2020). Activating transcription factor 6 mediates inflammatory signals in intestinal epithelial cells upon endoplasmic reticulum stress. Gastroenterology 159 (4), 1357–1374. e1310. 10.1053/j.gastro.2020.06.088 32673694 PMC7923714

[B33] Stoecklin-Wasmer C. Guarnieri P. Celenti R. Demmer R. T. Kebschull M. Papapanou P. N. (2012). MicroRNAs and their target genes in gingival tissues. J. Dent. Res. 91 (10), 934–940. 10.1177/0022034512456551 22879578 PMC3446831

[B34] Thuerauf D. J. Marcinko M. Belmont P. J. Glembotski C. C. (2007). Effects of the isoform-specific characteristics of ATF6 alpha and ATF6 beta on endoplasmic reticulum stress response gene expression and cell viability. J. Biol. Chem. 282 (31), 22865–22878. 10.1074/jbc.M701213200 17522056

[B35] Valadi H. Ekström K. Bossios A. Sjöstrand M. Lee J. J. Lötvall J. O. (2007). Exosome-mediated transfer of mRNAs and microRNAs is a novel mechanism of genetic exchange between cells. Nat. Cell Biol. 9 (6), 654–659. 10.1038/ncb1596 17486113

[B36] Watanabe T. Kukita T. Kukita A. Wada N. Toh K. Nagata K. (2004). Direct stimulation of osteoclastogenesis by MIP-1alpha: Evidence obtained from studies using RAW264 cell clone highly responsive to RANKL. J. Endocrinol. 180 (1), 193–201. 10.1677/joe.0.1800193 14709158

[B37] Watanabe Y. Fukuda T. Hayashi C. Nakao Y. Toyoda M. Kawakami K. (2022). Extracellular vesicles derived from GMSCs stimulated with TNF-α and IFN-α promote M2 macrophage polarization via enhanced CD73 and CD5L expression. Sci. Rep. 12 (1), 13344. 10.1038/s41598-022-17692-0 35922474 PMC9349189

[B38] Xia Y. Wei K. Hu L. Q. Zhou C. R. Lu Z. B. Zhan G. S. (2020). Exosome-mediated transfer of miR-1260b promotes cell invasion through Wnt/β-catenin signaling pathway in lung adenocarcinoma. J. Cell. Physiol. 235, 6843–6853. 10.1002/jcp.29578 32026462

[B39] Xiang L. Chen M. He L. Cai B. Du Y. Zhang X. (2014). Wnt5a regulates dental follicle stem/progenitor cells of the periodontium. Stem Cell Res. Ther. 5 (6), 135. 10.1186/scrt525 25510849 PMC4446079

[B40] Xiong Y. Chen H. Lin P. Wang A. Wang L. Jin Y. (2017). ATF6 knockdown decreases apoptosis, arrests the S phase of the cell cycle, and increases steroid hormone production in mouse granulosa cells. Am. J. Physiol. Cell Physiol. 312 (3), C341–C353. 10.1152/ajpcell.00222.2016 28100484

[B41] Yamada H. Nakajima T. Domon H. Honda T. Yamazaki K. (2015). Endoplasmic reticulum stress response and bone loss in experimental periodontitis in mice. J. Periodontal Res. 50 (4), 500–508. 10.1111/jre.12232 25223277

[B42] Zhang Q. Nguyen P. Xu Q. Park W. Lee S. Furuhashi A. (2017). Neural progenitor-like cells induced from human gingiva-derived mesenchymal stem cells regulate myelination of schwann cells in rat sciatic nerve regeneration. Stem Cells Transl. Med. 6 (2), 458–470. 10.5966/sctm.2016-0177 28191764 PMC5442816

