# Supplementary material for: miR-1260b inhibits periodontal bone loss by targeting ATF6β mediated regulation of ER stress
Source: Front Cell Dev Biol. 2022 Nov 30;10:1061216. doi: 10.3389/fcell.2022.1061216 (PMC9748617; doi:10.3389/fcell.2022.1061216)

Supplementary Material

# Supplementary Figure 1.

**Schematic illustration of the measurements for periodontal bone loss**

**(A)** Three-dimensional region of interest (ROI) and mesiodistal landmarks


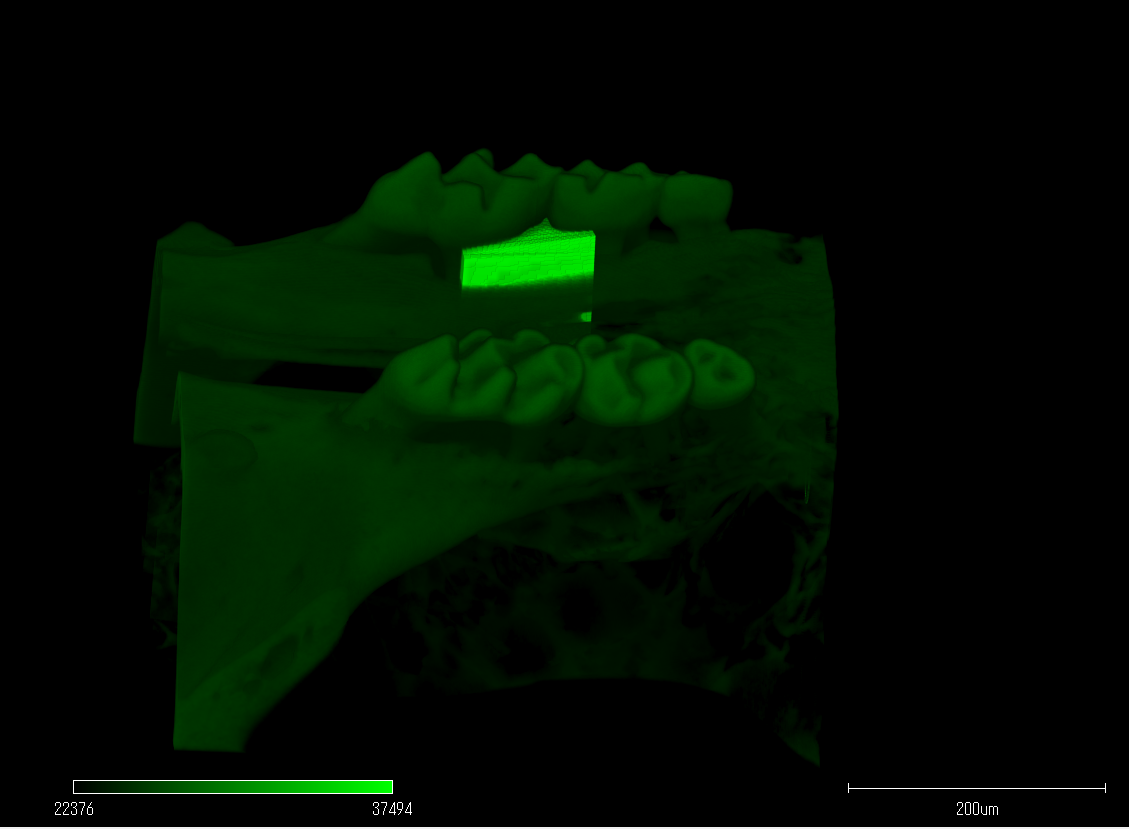

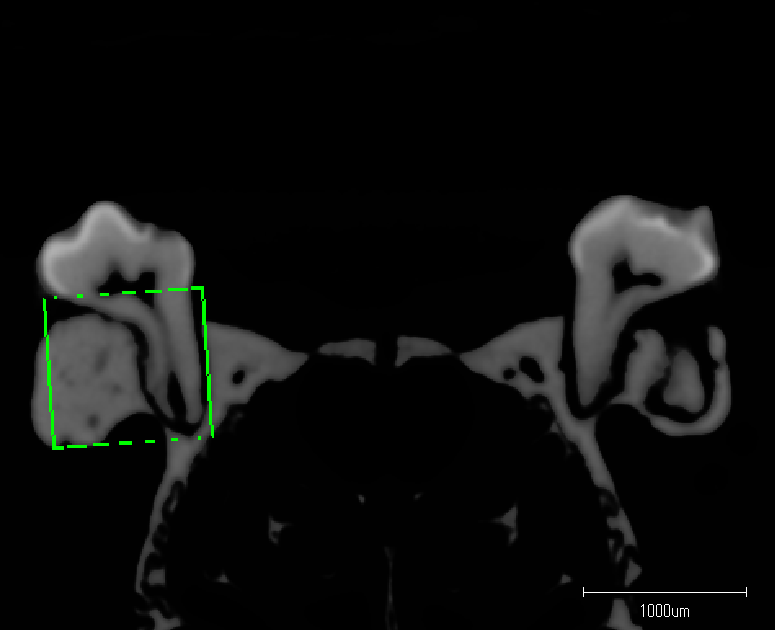

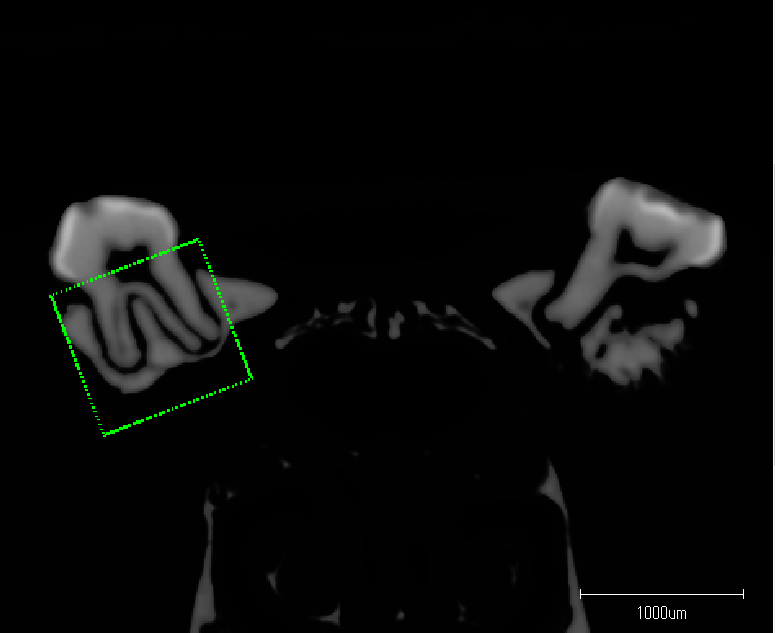


Mesial

Distal

**(B)** Distance from CEJ to AB


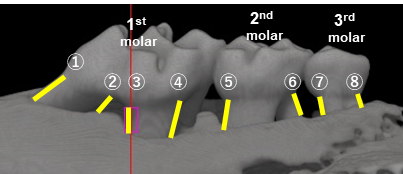


**Supplementary Table 1.**

Primer sequence used for quantitative RT-PCR

| **Gene** | **Forward primer** | **Reverse primer** |
| --- | --- | --- |
| ***human RANKL*** | 5'-**AGAGCGCAGATGGATCCTAA**-3' | 5'-**TTCCTTTTGCACAGCTCCTT**-3' |
| ***human ATF6β*** | **5’-CAGCACCTTGTATTCTGGCCT-3** | **5’-CCGTCAAACGGGACATCCT-3’** |
| ***human GAPDH*** | **5'-ATCAAGAAGGTGGTGAAGCAGG-3'** | **5'-GTCATACCAGGAAATGAGC-3'** |
| ***mouse ATF6β*** | **5'-TCTCCTCGGATGAGCAGGG-3'** | **5'-CTTCCCGAAGGGGTTCCAT-3'** |
| **mouse DC-STAMP** | **5'-AAAACCCTTGGGCTGTTCTT-3'** | **5'-AATCATGGACGACTCCTTGG -3'** |
| **mouse NFATc1** | **5'-GGAGAGTCCGAGAATCGAGAT-3'** | **5'-TTGCAGCTAGGAAGTACGTCT-3'** |
| **mouse TRAF6** | **5'-AGTGCCCAGTTGACAATGAAA-3'** | **5'-CACTTTACCGTCAGGGAAAGAAT-3'** |
| **mouse RANK** | **5'-AGTGCCCAGTTGACAATGAAA-3'** | **5'-CACTTTACCGTCAGGGAAAGAAT-3'** |
| ***mouse 18s rRNA*** | **5'-GCTTAATTTGACTCAACACGGGA-3'** | **5'-AGCTATCAATCTGTCAATCCTGTC -3'** |

# Supplementary Figure 2.

**Effects of ATF6β knockdown on osteoclast differentiation under ER stress in RAW-D cells**

RAW-D cells were transfected with si-Ctrl or si-ATF6β for 24 h. After changing the medium, cells were stimulated with RANKL (50 ng/mL) in the presence or absence of tunicamycin (0.5 μg/mL) for 24 h. The mRNA expressions of representative osteoclast differentiation marker for DC-STAMP, NFATc1, TRAF6 and RANK were measured by qRT-PCR. (n = 3). ns: not significant, *P < 0.05, ****P < 0.0001. The significance of differences between groups was determined using two-way ANOVA, followed by correction for multiple comparisons with Tukey’s post hoc test.


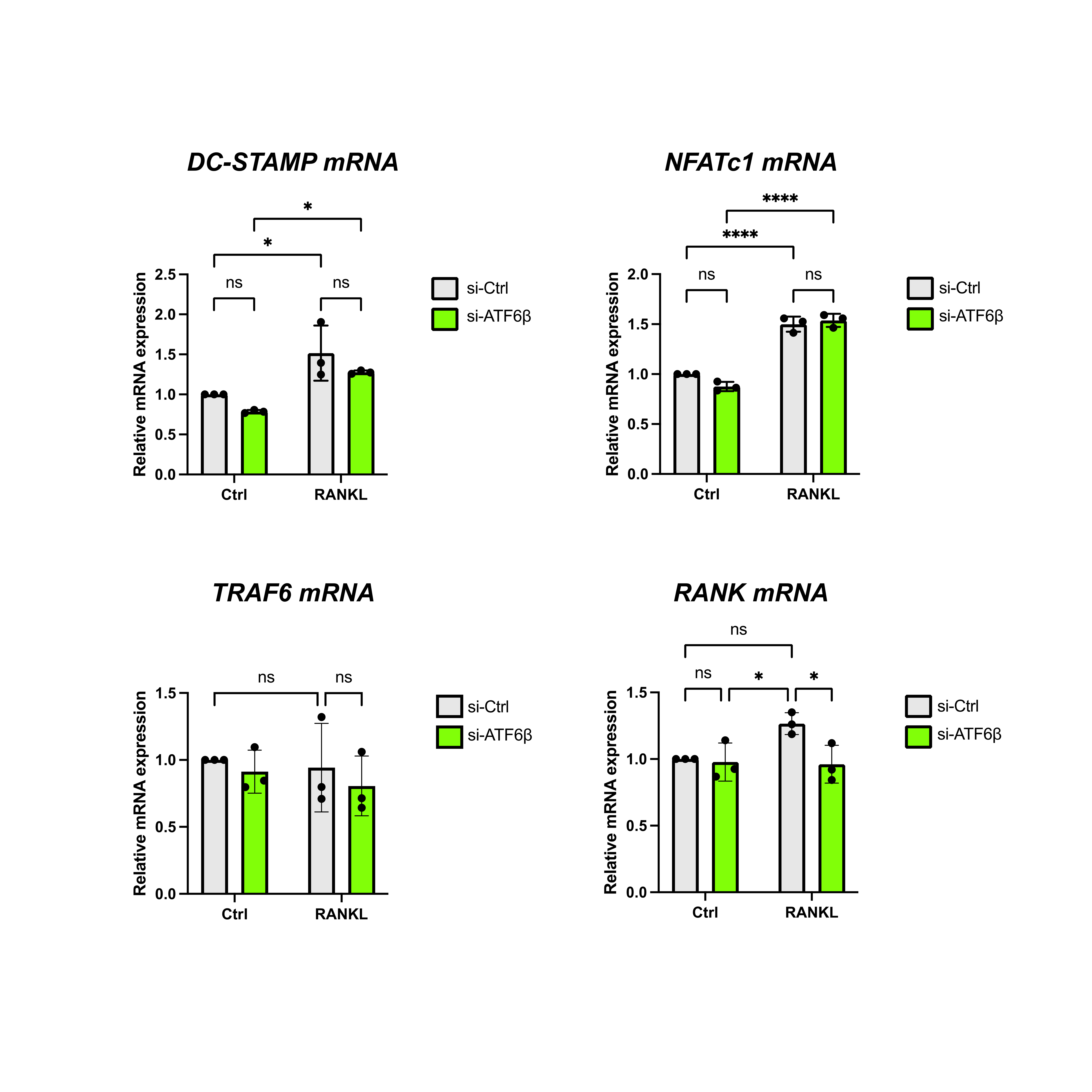

Supplement: Supplementary file 1 [file Table1.docx]
